# Early detection of perinatal depression in couples: a single-center prospective study

**DOI:** 10.1192/j.eurpsy.2024.1755

**Published:** 2024-09-03

**Authors:** Anne Paria, Anthony Atallah, Mikail Nourredine, Gil Dubernard, Fanny Joubert, Verena Landel, Sylvie Viaux-Savelon, Benoit De la Fournière

**Affiliations:** 1 Service de Gynécologie-Obstétrique, Hôpital de la Croix-Rousse, Hospices Civils de Lyon, Université Lyon 1 Claude Bernard, Lyon, France; 2 Service de Gynécologie-Obstétrique, Hôpital Femme Mère Enfant, Hospices Civils de Lyon, Université Lyon 1 Claude Bernard, Lyon, France; 3 Service de Biostatistiques, Hospices Civils de Lyon, Université Lyon 1 Claude Bernard, Lyon, France; 4 CRC GHN, Hôpital de la Croix Rousse, Hospices Civils de Lyon, Lyon, France; 5 Service de Relecture Scientifique, Hospices Civils de Lyon, Lyon, France; 6 Psy-perinatality Unit, Hôpital de la Croix Rousse, Hospices Civils de Lyon, Université Lyon 1 Claude Bernard, Lyon, France

**Keywords:** couple, perinatal depression, early postnatal visit, early prenatal visit

## Abstract

**Objective:**

This prospective study aimed to assess couples’ psychological status during the perinatal period to identify those at risk for postpartum depression.

**Methods:**

Conducted at Lyon University Hospital from March to July 2022, the study enrolled pregnant women without progressive psychiatric disorders or obstetric risk factors, and their partners. Participants completed the Edinburgh Postnatal Depression Scale (EPDS) at three points: during the 9th month of pregnancy, immediate postpartum, and 6–8 weeks after delivery. A score ≥10 on the EPDS indicated depression risk. A score ≥10 on the EPDS indicate depression risk. The primary endpoint was EPDS scores throughout the perinatal period.

**Results:**

Ninety-five couples participated; 96% of patients and 68% of partners completed pre-delivery questionnaires, 81% and 71% during maternity stay, and 64% and 46% postpartum, respectively. Overall, 15% of patients and 1% of partners had EPDS scores >10 in the postpartum period. Psychiatric history and emergency cesarean sections were associated with higher immediate postpartum EPDS scores in patients [Beta 3.7 points, 95% CI 0.91; 6.4 and Beta 5.2 points, 2.2; 8.1, respectively]. Episiotomy was associated with higher EPDS scores in partners. No significant association between the different factors studied and the EPDS score was found at 6–8 weeks postpartum in patients nor their partners.

**Conclusions:**

While specific risk factors for persistent perinatal depression in couples were not identified, a notable proportion of patients exhibited high EPDS scores. Screening all couples during prepartum and postpartum periods is crucial, regardless of identified risk factors.

## Introduction

Perinatal depression affects 10–15% of women worldwide [1]. In addition to the adverse health consequences for the mother, the most feared complication is death by suicide; in France, perinatal mental health disease and suicide are now the second cause of maternal death in the year following childbirth [2]. Moreover, maternal depression affects the whole family and impacts mother–infant bonding and the development of the child [3]. It is nowadays well established that infancy is a highly sensitive period for mental, motor, and emotional development. Such development is strongly dependent on environmental factors and particularly on the quality of early parent–child interaction [4], which can be impacted even in the case of mild depressive symptoms [5, 6].

Risk factors of perinatal depression have been studied and include personal or family psychiatric history, multiple exposures to difficult life events [7], lack of social support, history of traumatic pregnancy experience [8], extreme ages [9], and obstetrical factors [10].

Recent studies highlight also the impact of father’s postpartum depression, estimated between 8 and 10% in France [11] on the mother’s psychological status [12]. Such findings have led to a paradigm shift: clinicians and researchers now prefer to focus on perinatal parental depression rather than on maternal postpartum depression only [13]. But studies with both mother and father data collected together are still scarce.

Most studies conclude that there is a need to detect perinatal depression as early and as widely as possible in order to limit its consequences for the mother, the couple, the child’s development, and the mother–infant relationship.

Several scales have been developed for this purpose; the most commonly used is the Edinburgh Postnatal Depression Scale (EPDS) [14], which has also been validated during pregnancy [15] and in men [11]. In France, recent recommendations require professionals to systematically propose early prenatal [16] and postnatal interviews [17]. These interviews must be offered to both members of the couple and aim to detect risk factors and first symptoms of depression, in order to refer them for appropriate follow-up. However, there are few prospective studies testing the feasibility of the couple depression screening in prepartum and postpartum and there is still no consensus on the best tool nor on the time frame for perinatal depression screening [18]. Given the lack of data regarding the psychological status of couples throughout the perinatal period, there is a need for longitudinal prospective analyses assessing the mood disorders of both parents during the perinatal period.

The present study aims to prospectively assess the depressive symptoms of couples through the EPDS questionnaire during the prenatal, immediate and late postpartum periods in a low-risk population in order to identify parents at increased risk for postpartum depression.

## Materials and methods

### Study design and participants

We conducted a single-center observational prospective study based on the 2020 and 2022 recommendations of the French National Authority for Health (Haute Autorité de Santé) [16, 17]. All couples followed up between March 7 and May 13, 2022 at the Croix Rousse maternity of the Lyon University Hospital, France, and who agreed to participate, were asked to fill the EPDS at three different endpoints: the 9th month pregnancy consultation, during the stay in the maternity ward after delivery (immediate postpartum period), and between 6 and 8 weeks after delivery (late postpartum period).

The study was conducted in accordance with the Declaration of Helsinki and was approved by the local ethics committee (number 22-51790). As observational study according to National Health Care Recommendations, all participants received an information leaflet detailing the protocol and the collected information but did not have to give written consent.

The inclusion criteria were: pregnant women over 18 years of age and their partners during the 9th month of pregnancy, with a pregnancy follow-up and delivery at the Croix Rousse maternity. Patients and partners that did not speak French, or that were treated for an ongoing psychiatric or psychological disorder, at the time of the inclusion, were excluded.

Eligible patients and their partners were recruited by the healthcare professionals providing the 9th month consultation (physician or midwife) between 37 and 39 weeks of gestation. The two first EPDS were filled during the antenatal care consultation and during the maternity stay. The third EPDS questionnaire was collected by email or phone for the late postpartum period.

The threshold of the EPDS test varies depending on the studies. For women, it ranges from 5/6 to 13, depending on the desired sensitivity and specificity [19–21]. For men, it is often around 9 [22]. We aimed to use the same threshold for both members of the couple. In a screening approach and to achieve the best sensitivity, as in the following studies, we opted for a threshold of 10 on the EPDS. This threshold is also found in the literature for men and has been validated by the French National Authority for Health (HAS) for perinatal depression screening in the French population [23].

### Data collection

Sociodemographic and medical data were collected at inclusion from the patients’ medical records. Data about delivery were collected after delivery, before the last EPDS score collection. EPDS scores were collected at the 9th month of pregnancy, during the maternity stay, and 6–8 weeks postpartum. The EPDS self-questionnaire was completed at the hospital for the antenatal period and during the maternity stay. It was completed from home for the delayed postpartum period, by email or telephone. For patients who did not respond to the first email, three attempts by telephone and two additional attempts by email were made.

Data were collected from the medical record and analyzed by the medical team leading the project (A.P., S.V.S., and B.D.F.).

The EPDS is a 10-item postnatal questionnaire that has been validated as a depression screening tool throughout the perinatal period, translated and validated in French [24]. The EPDS score ranges from 0 to 30. A cut-off value of 10 or higher was defined to qualify a risk of perinatal depression [20, 25, 26]. A psychological or psychiatric medical support was offered to the patients presenting during the study period at least one score higher or equal to 10. In these cases, patients were offered psychological and psychiatric follow-up either by the hospital team or after referral to perinatal healthcare professionals close to their home.

### Statistical analysis

The usual position and dispersion parameters were employed to describe the quantitative and qualitative data.

A mixed model assessing the effect of primiparity on the evolution of the EPDS score over time was performed. The model included as a random effect: an intercept and a slope; as a fixed effect: age, time, primiparity, and a quadratic effect over time. The association between different clinical factors and the EPDS score at different times was estimated by mean differences with their confidence intervals.

The correlation between each pair of time combination of EPDS scores was estimated by a Pearson correlation coefficient and its 95% confidence interval.

Analyses were performed using R® software version 4.1.2.

## Results

### General characteristics of the study population

**Figure 1.**
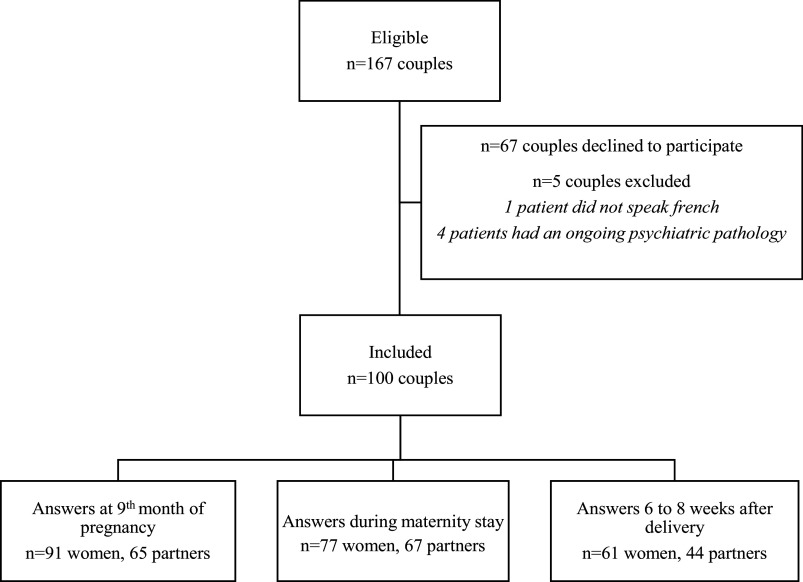
Study flow chart.

Overall, 167 couples were eligible but 100 agreed to participate; 5 couples were excluded, 1 because the couple did not speak French and 4 because the patients had ongoing psychiatric pathology or were under antidepressant treatment (Figure 1). At inclusion, the mean age was 32.7 years for the patients and 34.4 years for their partners. Most of the patients (*n* = 41/95; 43%) and their partners (*n* = 43/95; 45%) occupied higher intellectual professions, based on employment status. A personal history of psychiatric disease was reported by 13/95 (14%) patients and 2/95 (2%) partners. Among the studied population, 53% (*n* = 50/95) accepted and underwent mandatory early perinatal counseling, held between 14 and 20 weeks of gestation (Table 1).

**Table 1.** Characteristics of the population

Characteristics	Patient, *N* = 95^a^	Partner, *N* = 95^a^
Age	32.7 (4.7)	34.4 (5.3)
Profession		
High socioeconomic status	41 (43%)	43 (45%)
Intermediate professions	33 (35%)	23 (24%)
Employees	11 (12%)	13 (14%)
Craftsmen, shopkeepers, company managers	3 (3.2%)	12 (13%)
Workers	1 (1.1%)	3 (3%)
Others and unemployed	6 (6.3%)	1 (1%)
History of psychological or psychiatric follow–up	13 (14%)	2 (2%)
ART	10 (11%)	10 (11%)
Early prenatal interview		
Yes	50 (53%)	15 (16%)
No	26 (27%)	39 (41%)
Unknown	19 (20%)	41 (43%)
EPDS score		
At the 9th month of pregnancy	6.0 (4.7)	3.4 (3.1)
During the maternity stay	5.4 (4.4)	3.1 (3.1)
6–8 weeks postpartum	5.8 (6.1)	2.1 (2.8)
Mean response delay in the postpartum period (days)	58 (22)	60 (23)

Abbreviations: ART, assisted reproductive therapy; EPDS, Edinburgh Postnatal Depression Scale.

a Mean (SD); *n* (%).

**Figure 2.**
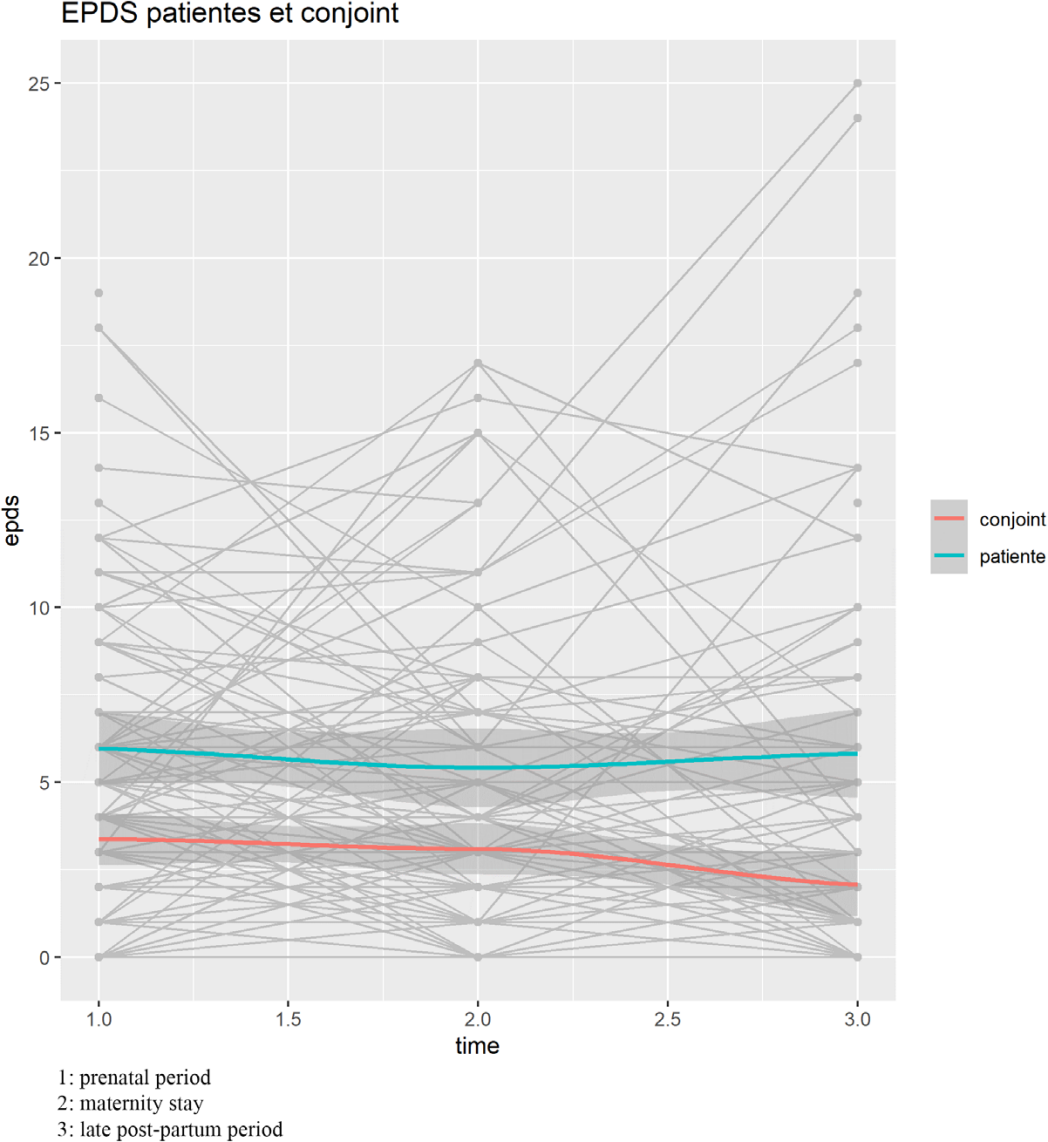
Changes in the EPDS score over time.

Overall, 91/95 (96%) patients and 65/95 (68%) partners answered the EPDS questionnaire at the 9th month of pregnancy, 77/95 (81%) and 67/95 (71%) during the maternity stay, and 61/95 (64%) and 44/95 (46%) in the postpartum period. At the 9th month of pregnancy, the mean EPDS score was 6/30 (SD 4.7) for the patients and 3.4/30 (SD 3.1) for their partners. It was 5.4/30 (SD 4.4) and 3.1/30 (SD 3.1) during the maternity stay, then 5.8/30 (SD 6.1) and 2.1/30 (SD 2.8) in the late postpartum period (Figure 2).

Overall, 14 patients and 1 partner had an EPDS score ≥ 10 in the late postpartum period; they were all contacted and referred for consultation with the hospital psychiatric team or to an outpatient psychological and psychiatric care near their home. One patient was followed by the psychiatric team of the maternity hospital.

### Obstetrical data and perinatal outcomes

Half of the patients were primiparous (*n* = 48/95); 23% (*n* = 22/95) had already had at least one miscarriage, and 1% (1/95) had undergone a medical termination of pregnancy (Table 2). None had experienced intrauterine fetal demise. Among the multiparous women, one (2%) had a history of pre-eclampsia and one (2%) had been followed up for intrauterine growth restriction during a previous pregnancy. The previous pregnancies had all led to full-term deliveries except one (2%) that delivered at 36 + 1 weeks of gestation; 38% (18/47) had a history of medical intervention during a previous delivery (i.e., personal history of induction of labor and/or assisted vaginal delivery and/or cesarean section and/or hemorrhage) (Table 2).

**Table 2.** Obstetrical history, pregnancy course, and delivery modalities

Characteristics	*N* = 95^a^
BMI (kg/m^2^) (standard deviation)	22.0 (3.9)
Parity	
0	48 (50%)
1	37 (39%)
**≥**2	10 (11%)
History of pathological pregnancy	3 (3%)
History of miscarriage	
0	73 (77%)
1	17 (18%)
**≥**2	5 (5%)
History of ectopic pregnancy	2 (2%)
History of medical intervention during delivery	18 (38%)
Gestational diabetes	13 (14%)
Pregnancy follow–up at the fetal anomaly clinic	12 (13%)
Labor initiation	
Spontaneous	69 (73%)
Medical induction	23 (24%)
Cesarean section before labor (standard deviation)	3 (3%)
Mean labor time on the partogram	5.0 (4.2)
Epidural anesthesia	
Yes	83 (87%)
No, because unwanted	6 (6.5%)
No, because of precipitous labor	6 (6.5%)
Delivery modality	
Spontaneous vaginal delivery	66 (69.5%)
Assisted vaginal delivery	17 (18%)
Cesarean section before labor	3 (3%)
Emergency cesarean section	9 (9.5%)
Emergency code	
Green	14 (54%)
Orange	5 (19%)
Red	7 (27%)
Perineal tear degree	
Intact	35 (37%)
1st degree	32 (33.5%)
2nd degree	20 (21%)
3rd degree	2 (2%)
Episiotomy	6 (6.5%)
Hemorrhage	8 (8%)
Birth weight (g) (standard deviation)	3400 (389)
Arterial pH Apgar at 1 min	7.26 (0.08)
**≤**7	9 (9%)
8	10 (11%)
9	14 (15%)
10	62 (65%)
Apgar at 5 min	
**≤**8	2 (2%)
9	10 (11%)
10	83 (87%)
Feeding type	
Formula	15 (16%)
Breastfeeding	78 (82%)
Mixed	2 (2%)

a Median (SD); *n* (%).

Overall, 76% (*n* = 72/95) of patients had uneventful pregnancies, except for 14% (*n* = 13/95) that presented gestational diabetes and 13% (*n* = 12/95) that were referred to the fetal anomaly clinic. Among those patients, no significant fetal malformation had been described. Nine patients (9%) accepted a psychological follow-up during their pregnancies.

Overall, 69/95 (73%) patients went into spontaneous labor and 66/95 (69%) had normal spontaneous vaginal deliveries. During labor, 6/95 patients (6.5%) wished but did not receive an epidural anesthesia, due to technical and organizational issues. Among the assisted deliveries and nonelective cesarean sections, 54% (*n* = 14/26) were nonurgent (code green, delivery recommended within an hour), 19% (*n* = 5/26) were relatively urgent (code orange, delivery recommended within 30 min), and 27% (*n* = 7/26) were very urgent (code red, delivery recommended within 15 min). Eight patients (8%) were diagnosed with postpartum hemorrhage (bleeding ≥500 mL), requiring obstetrical intervention.

The neonates had a normal weight, except 2/95 (2%) of them who were below the 3rd percentile according to the AUDIPOG curve [27]. Their umbilical cord pH was also within norms, only one arterial pH was below 7.10 in the context of an emergency vacuum birth for fetal bradycardia. Only one baby (*n* = 1/95; 1%) required a transfer to the neonatology ward for respiratory distress, with a length of stay of 7 days.

### Perinatal EPDS score according to patients and partners characteristics

**Figure 3.**
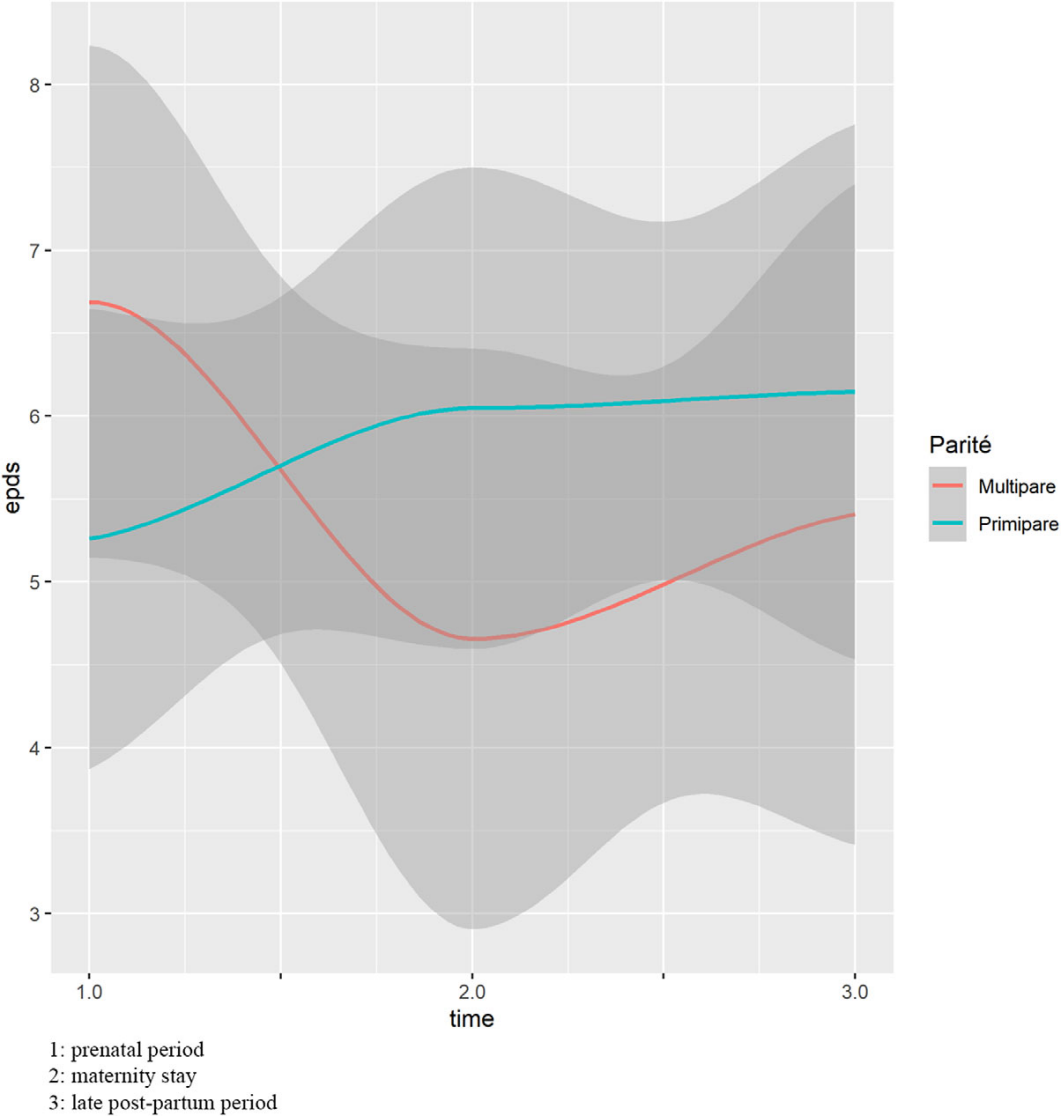
Effect of parity on the changes in EPDS score over time among patients (mixed model).

Among the patients, a personal history of psychiatric disease was associated with an increase in the EPDS score (3.7 points [95% CI 0.91; 6.4]) during the maternity stay (Table 3). According to the mixed model, at constant age, primiparity seemed to increase non-significantly the EPDS score by 1.02 [95% CI −0.40; 2.44] (Figure 3).

**Table 3.** EPDS score variations according to patients’ characteristics

	EPDS *t*1		EPDS *t*2		EPDS *t*3	
Characteristics	Beta	95% CI	Beta	95% CI	Beta	95% CI
Age	−0.01	−0.22, 0.20	−0.02	−0.26, 0.22	−0.24	−0.59, 0.11
Profession						
Craftsmen, shopkeepers, company managers	–	–	–	–	–	–
Executives and higher intellectual professions	−1.0	−6.5, 4.5	0.11	−5.3, 5.5	2.0	−5.5, 9.4
Intermediate professions	−1.6	−7.2, 3.9	0.32	−5.2, 5.8	1.3	−6.5, 9.1
Employees	2.1	−3.9, 8.0	−0.76	−7.0, 5.5	4.3	−4.0, 13
Others and unemployed	0.83	−5.6, 7.3	−1.3	−12, 9.1	−0.67	−15, 14
BMI	0.00	−0.25, 0.24	0.17	−6.7, 7.1	6.8	−4.4, 18
Parity	0.60	−0.90, 2.1	−1.0	−2.4, 0.41	−0.60	−3.0, 1.8
History of miscarriage	0.45	−1.2, 2.1	−0.03	−1.9, 1.9	−3.0	−6.0, 0.00
History of medical intervention during delivery						
No	–	–	–	–	–	–
Yes	−0.48	−3.8, 2.8	−0.49	−3.1, 2.1	−3.6	−8.9, 1.7
History of psychological or psychiatric follow–up						
No	–	–	–	–	–	–
Yes	0.76	−2.0, 3.5	3.7	0.91, 6.4	2.3	−2.1, 6.7
ART						
No	–	–	–	–	–	–
Yes	−1.4	−4.5, 1.7	−1.7	−5.2, 1.7	−3.5	−8.7, 1.7
Early prenatal interview						
No	–	–	–	–	–	–
Yes	−0.71	−3.1, 1.7	0.43	−2.1, 3.0	−1.5	−5.1, 2.1
Gestational diabetes						
No	–	–	–	–	–	–
Yes	−0.44	−3.3, 2.4	−0.49	−3.3, 2.3	0.08	−4.3, 4.5
Follow–up at the antenatal diagnosis center						
No	–	–	–	–	–	–
Yes	0.66	−2.3, 3.6	1.7	−1.4, 4.8	2.4	−1.9, 6.8

Abbreviations: ART, assisted reproductive therapy; BMI, body mass index; CI, confidence interval; *t*1, 9th month consultation; *t*2, maternity stay; *t*3, 6–8 weeks postpartum.

Among the partners, no significant association was found between the EPDS score and the studied factors (Table 4).

**Table 4.** Partners EPDS score variations over time

	EPDS *t*1		EPDS *t*2		EPDS *t*3	
Characteristics	Beta	95% CI	Beta	95% CI	Beta	95% CI
Age	−0.06	−0.20, 0.08	−0.13	−0.27, 0.02	−0.10	−0.27, 0.07
Profession						
Craftsmen, shopkeepers, company managers	–	–	–	–	–	–
Executives and higher intellectual professions	0.40	−2.6, 3.4	−1.2	−4.3, 1.8	−0.77	−5.0, 3.5
Intermediate professions	1.5	−1.7, 4.7	0.51	−2.7, 3.7	0.19	−4.2, 4.6
Employees	1.0	−3.0, 5.0	−0.23	−3.8, 3.4	0.00	−5.8, 5.8
Workers	1.9	−3.3, 7.1	−0.60	−7.5, 6.3	−1.5	−8.6, 5.6
History of psychological or psychiatric follow–up						
No	–	–	–	–	–	–
Yes	−3.4	−9.6, 2.8	0.42	−4.1, 5.0	−2.1	−7.8, 3.6
ART						
No	–	–	–	–	–	–
Yes	1.6	−0.74, 3.9	−0.28	−3.0, 2.4	−0.85	−3.3, 1.6
Early prenatal interview						
No	–	–	–	–	–	–
Yes	0.57	−1.5, 2.6	−0.75	−3.2, 1.7	−1.1	−3.4, 1.1
Follow–up at the antenatal diagnosis center						
No	–	–	–	–	–	–
Yes	−0.28	−2.6, 2.1	0.86	−1.7, 3.4	1.1	−1.4, 3.5

Abbreviations: CI, confidence interval; *t*1, 9th month consultation; *t*2, maternity stay; *t*3, 6–8 weeks postpartum.

### Impact of delivery mode on immediate and late EPDS score

Among the patients, the onset of an emergency cesarean section significantly increased the EPDS score by 5.2 [95% CI 2.2; 8.1] points in the immediate postpartum period compared with a spontaneous vaginal delivery (Table 5). An elective cesarean section was correlated with a non-significant increase of the patients EPDS score (2.3 [95% CI −6.1; 11]) in the immediate postpartum period and a non-significant decrease of the partner EPDS score in both the immediate (−1.4 [95% CI −7.8; 5.0]) and delayed postpartum period (−2.0 [95% CI −6.1; 2.1]).

**Table 5.** Influence of labor course and delivery on the EPDS scores of the patients and theirs partners

	Patient				Partner			
	EPDS *t*2		EPDS *t*3		EPDS *t*2		EPDS *t*3	
Characteristics	Beta	95% CI	Beta	95% CI	Beta	95% CI	Beta	95% CI
Labor initiation								
Spontaneous	–	–	–	–	–	–	–	–
Medical induction	0.44	−1.8, 2.7	2.4	−1.1, 5.8	0.12	−1.7, 2.0	−0.44	−2.4, 1.5
Cesarean section before labor	1.7	−7.2, 11	1.4	−7.4, 10	−1.1	−7.5, 5.4	−1.8	−5.9, 2.4
Mean labor time	0.04	−0.21, 0.29	−0.31	−0.73, 0.11	0.01	−0.22, 0.24	−0.05	−0.36, 0.26
Epidural anesthesia								
Yes	–	–	–	–	–	–	–	–
No, because unwanted	−1.9	−5.9, 2.1	−3.3	−11, 4.0	0.83	−2.1, 3.7	−1.6	−4.9, 1.8
No, because of precipitous labor	−2.5	−6.5, 1.5	0.54	−8.3, 9.3	−2.4	−5.6, 0.80	−2.2	−7.9, 3.5
Delivery modality								
Spontaneous vaginal birth	–	–	–	–	–	–	–	–
Cesarean section before labor	2.3	−6.1, 11	0.87	−8.1, 9.8	−1.4	−7.8, 5.0	−2.0	−6.1, 2.1
Emergency cesarean section	5.2	2.2, 8.1	1.9	−3.1, 7.0	−0.38	−3.4, 2.6	0.02	−3.0, 3.0
Extraction	0.27	−2.1, 2.7	−0.27	−4.1, 3.6	−1.2	−3.1, 0.76	−1.3	−3.3, 0.71
Emergency degree								
Code green	–	–	–	–	–	–	–	–
Code orange	3.2	−2.0, 8.4	4.7	−3.8, 13	0.53	−3.7, 4.7	−0.10	−4.0, 3.8
Code red	1.3	−3.5, 6.2	2.8	−3.4, 9.0	−0.17	−3.9, 3.5	−0.06	−3.7, 3.6
Perineal tear degree								
Intact	–	–	–	–	–	–	–	–
1st degree	−0.85	−3.3, 1.6	0.86	−3.0, 4.7	1.2	−0.61, 2.9	0.44	−1.8, 2.6
2nd degree	−2.8	−5.5, −0.17	−0.39	−4.7, 3.9	−0.29	−2.2, 1.6	−0.36	−2.7, 2.0
3rd degree	0.07	−6.3, 6.4	−4.8	−14, 4.4	3.1	−1.3, 7.4	−1.1	−5.4, 3.3
Episiotomy	−1.6	−5.5, 2.3	0.23	−5.9, 6.4	4.8	1.6, 8.0	0.27	−3.4, 3.9
Hemorrhage								
No	–	–	–	–	–	–	–	–
Yes	0.45	−3.3, 4.2	0.41	−5.3, 6.1	−0.89	−4.1, 2.4	−2.0	−4.9, 0.89
Apgar 1 min	0.04	−0.66, 0.75	0.74	−0.27, 1.8	−0.10	−0.63, 0.44	0.24	−0.29, 0.77
Apgar 5 min	−0.51	−3.8, 2.8	−0.76	−5.0, 3.5	−0.08	−2.8, 2.6	1.2	−0.77, 3.2

Abbreviations: CI, confidence interval; *t*2, maternity stay; *t*3, 6–8 weeks postpartum.

In patients, second degree perineal tears were significantly associated with lower EPDS scores in the immediate postpartum period (−2.8 [95% CI −5.5; −0.17]).

The performance of an episiotomy was the only factor to be significantly associated with the EPDS score in the partners, the latter increasing the EPDS score in the immediate postpartum period by 4.8 points [95% CI 1.6; 8].

The absence of epidural seemed to be associated with a lower patient EPDS score in the immediate postpartum period for both patients who did not want it (−1.9 [95% CI −5.9; 2.1]) and those who had a precipitous labor (−2.5 [95% CI −6.5; 1.5]). For the former patients, the score seemed to decrease further in the late postpartum period while it increased for the latter (Table 5).

### Correlation between EPDS scores at the three study time points

The correlation coefficients between the EPDS scores at the three time points were calculated in order to assess the intensity of the interdependence between score values and were as follows: rEPDS *t*1–*t*2 = 0.4 [0.2–0.58], rEPDS *t*2–*t*3 = 0.52 [0.3–0.69], rEPDS *t*1–*t*3 = 0.4 [0.15–0.59].

## Discussion

### Main findings

We prospectively studied the evolution of EPDS scores in both couple members, following the latest National recommendations. This assessment of both parents was repeated at three key time points during the perinatal period: at the 9th month of pregnancy, at birth, and after 2 months of the child’s life. To the best of our knowledge, there are no studies that have applied these new recommendations and demonstrated the feasibility of this assessment in both parents, as we have done. In the present population, 15% of patients and 1% of their partners had an EPDS score suggestive of postpartum depression 2 months after birth. The result for the patients is in accordance with the 2022 French national survey on maternal mortality [2], while that of the partners is lower than previously reported [11]. However, our study did not identify specific predictive factors for persistent perinatal depression among couples 6–8 weeks after childbirth in a low-risk population as illustrate in Figure 2. It underscores the importance of screening all couples during both the prepartum and postpartum periods, regardless of the presence of known risk factors. The suggestion to conduct repeated screenings for both couple members during three critical periods – prepartum, immediate postnatal, and remote postnatal – is considered innovative. This approach may enhance the likelihood of identifying individuals at high risk for perinatal depression.

### Antenatal risk factors

In this low-risk study population with no specific obstetrical nor social risk factor, no predictive factor for depressive symptoms in the late postpartum period was identified, despite a relatively large prevalence of patients with a EPDS score of 10 or higher. However, a personal history of psychiatric or psychological follow-up seems to be correlated with an increase in EPDS score during the late postpartum period, suggesting that this factor should be considered during the prenatal interview, in both members of the couple. Several authors have described personal or family history of psychiatric disorders as major risk factors for developing perinatal depression [9, 11, 28, 29]. However, this hypothesis is not unanimous, and may differ between men and women as reported by a recent meta-analysis [30] and as suggested by the present results. It is likely that the lack of association between a history of psychiatric or psychological follow-up and the EPDS score in the partners herein was due to the small proportion of partners with depressive symptoms and to the fact that the majority of partners did not spontaneously report this type of history during the initial consultation. According to previous findings, paternal perinatal depressive symptoms differ from those observed in women. Indeed, anger, irritability, hyperactivity, and poor impulse control are more common among men facing postpartum depressive symptoms [11]. Moreover, coping strategies are also divergent, with a greater propensity for isolation and drug or alcohol consumption in men than in women [31]. Thus, we suggest that postpartum depression might be under-diagnosed in partners and EPDS score could not be the best tool to screen such a condition. Plus, since domestic violence is increasingly highlighted during pregnancy follow-up consultations [32], developing new screening tools with evaluation of the patient vulnerability ought to be considered in future research [9].

Interestingly, although it was not statistically significant, our results show a progressive decrease in EPDS score over time, in both members of the couple, when pregnancy was obtained following assisted reproductive therapy. This is discordant with studies showing that couples going through this long and stressful process are more likely to develop anxious manifestations that disturb the establishment of a privileged link with their child [33–35]. Some authors have hypothesized that the anxiety or depressive symptoms in these parents may be due to a longer period of grief regarding the idealized child [36]. However, not all studies report depressive symptoms in these couples [37–39], and the present results tend to suggest a potential protective effect of ART toward perinatal depression, possibly explained by the achievement of a well-considered project, and the strong investment of both members of the couple.

Conversely, pregnancy monitoring in the fetal anomaly clinic seemed to gradually increase the EPDS score over time in both members of the couple. It is possible that couples apprehend better the last month of the pregnancy compared to earlier months when a higher number of stressful appointments and achievements can occur, leading to difficult decisions such as undertaking amniocentesis or requesting a termination of pregnancy. It is worth mentioning that the fetuses followed up in the fetal anomaly clinic herein did not carry serious anomalies that could have led to a request for a medical termination of the pregnancy. However, the anxiety generated by these additional appointments may generate ambivalent psychological state in both parents at birth or during the weeks after. Although few studies have addressed this subject, a recent one reported increased postpartum anxiety and depression in patients followed up for fetal anomaly or abnormal ultrasound findings [40].

When evaluating the effect of parity on the EPDS score independently of age, no significant association between EPDS score over time and parity was observed. The EPDS score seemed to be higher in multiparous women in the antenatal period, then the two curves crossed at delivery, and the score of the multiparous women then tended to be lower in the immediate and late postpartum periods (Figure 3). This observation is consistent with the literature [41–43], and can be explained by the accumulated tiredness of multiparous women at the end of pregnancy, and by the inexperience of primiparous women who are confronted for the first time with labor and delivery, and then have to learn how to manage the needs of a newborn.

Of note, only half of the patients and very few partners herein had received early pregnancy prenatal counseling, appointment recommended by National Health Care Recommendations thus preventing a clear evaluation of the effect of this clinic on the risk of postpartum depression. Since the aim of this counseling is to identify the needs in terms of support during pregnancy [44], efforts must continue to offer the pregnancy counseling to both members of the couple.

### Delivery-related risk factors

Our study highlights a significant prevalence of high EPDS scores during their maternity stay among patients with a personal history of psychological disorders and those who underwent emergency cesarean sections that could be linked to acute post traumatic symptoms. None factor related to the delivery was significantly associated with a change in the EPDS score at 6–8 weeks postpartum. However, the experience of both parents seems to differ in case of a cesarean section: the maternal EPDS score tended to increase in the immediate and late postpartum period, even more so in case of an emergency cesarean section. In opposition to vaginal delivery, during which endogenous oxytocin discharge is higher [45, 46], and skin-to-skin contact is possible, it has been suggested that the surgical environment could trigger postpartum depression [47]. Moreover, a Slovakian study published in 2021 showed that dissatisfaction with the birth process is a source of postpartum depression [48], and a Canadian study has recently shown that emergency cesarean section was indirectly linked to postpartum depression via a post-traumatic stress mechanism [49], Conversely, scheduling an elective cesarean section tended to be associated with a decrease in the partners’ EPDS scores, according to the present findings. To the best of our knowledge, this has not yet been described in the literature and should be further investigated. One possible explanation could be that the scheduling of the birth date may allow them to anticipate the constraints related to their professional and/or family life and to ensure their presence on this important day. Another hypothesis may be related to the difficulty felt by some partners regarding their role during labor and delivery. Indeed, if their presence is often considered as normal in some culture, it generates levels of vulnerability, especially regarding their partner’s pain, the shared deception of a long and unpredictable cervical dilation, and the role of a powerless spectator of their partner and baby’s fate. Finally, in spite of all the factors mentioned above, it is commonly expected for them to be cheerful and supportive. Thus, it seems essential to prepare partners for labor and delivery, and to support them throughout their journey in the delivery ward.

In case of vaginal delivery, no association was found between assisted delivery and the psychological status of women or partners in the immediate or late postpartum period. This is consistent with a large cohort study in Great Britain [50]. However, the degree of perineal tear was correlated with the EPDS score during maternity stay. Although the absence of perineal tear was not a protective factor, second degree tears was associated with a decrease in the maternal EPDS score. This surprising result, which concerned a majority of primiparous women, could be explained by the relief of not having endured an episiotomy. Since this change in the EPDS score was not observed later in the postpartum period, the results are consistent with the study of Kaya et al., that showed no difference in the maternal EPDS score at 1 and 3 months postpartum, according to whether or not an episiotomy was performed [51]. Conversely, the partners’ EPDS score seemed to increase non-significantly with the degree of perineal tear, and the performance of an episiotomy was associated with a significant increase in the EPDS score during the maternity stay, which did not persist 6–8 weeks later. This result, although not previously reported, is probably multifactorial. Episiotomies are often performed in emergency, in the context of instrumental delivery or fetal heart rate abnormalities [52, 53]. Although the patient’s consent is always sought, the context is not conducive to obtaining informed consent. At a time when mistrust of the medical profession is unprecedented, it is understandable that some partners may perceive this act as a potential mutilation. Furthermore, numerous studies have shown that episiotomy is correlated to persistent perineal pain in the postpartum period [54] and a delayed sexual health recovery without adverse sexual functions [55].

The results about analgesia during labor and delivery are consistent with numerous studies published on the link between pain, post-traumatic stress, and depression [56, 57]. The present results corroborate the well-established hypothesis that the perception of pain is multifactorial [58]. Patients who were prepared to manage the pain during labor and delivery and who chose not to have an epidural, had a decreased EPDS score during early and late postpartum period. This suggests that pain, in this case, did not lead to post-traumatic stress or mood changes. However, the mothers who did not receive an epidural anesthetic although they wished for it seemed to have a low EPDS score in the immediate postpartum period, probably linked to a rapid and eutocic delivery, but their score increased by three points 6–8 weeks later. This result highlights the time frame of post-traumatic stress and the depression [59, 60]. In the future, patients should be prepared for the possible lack of epidural anesthesia in the event of precipitous labor. The management of pain with non-pharmacological approaches, intravenous morphine or nitric oxide inhalation should be considered when the setting of an epidural is not possible.

Finally, the occurrence of a hemorrhage did not significantly impact the maternal EPDS score after delivery, which is consistent with a recent Swedish study [61]. The downward trend of the partners’ EPDS score in case of an abnormal bleeding could be explained by the same decrease observed in the case of a cesarean section, which is more prone to bleeding.

### Correlation between EPDS scores at the three time points

The EPDS scores at the three time points of the study were related to each other by a moderate positive correlation. Indeed, the EPDS scores in the antenatal and early postnatal periods were only slightly predictive of the EPDS score a few weeks after birth. Thus, the assessment of the mental health status of the couples during pregnancy and immediately after birth is important, as it allows early implementation of psychological or psychiatric care, but is not sufficient. Therefore, prevention and screening of mood disorders must be pursued after delivery as recommended by the French national health authority [15].

### Strengths and limitations

The major strength of this study is its prospective aspect. Moreover, the prevalence of patients with a score suggestive of depression is consistent with the literature [1, 62]. Importantly, psychological support was offered to patients when the score was 10 or greater and also when the score was normal but the caregivers felt that it could be beneficial. Overall, the offer of psychological or psychiatric counseling was well accepted and the patients took the initiative to contact the medical teams when advised to do so.

An important limitation of this study is the lack of power due to a high prevalence of lost to follow-up during the late postpartum period, despite numerous reminders. More than half of the partners did not answer the EPDS questionnaire in the weeks following the delivery, which prevented us to carry out all the statistical analyses initially planned. Moreover, the EPDS does not allow to diagnose depression *per se*, and as such it is possible that some patients with a high EPDS score were referred to a specialist consultation without any real indication for depression while others were not although they might have needed it. Since we did not have access to the contents of the psychological and psychiatric consultations, we do not have information on the prevalence of patients who were finally diagnosed with perinatal depression. Finally, the prevalence of an EPDS score over 10 in men was lower than expected, questioning the acceptance, timing, performance, validation, and selected cut-off in this population.

## Conclusion

This pioneering study aligned with the latest government recommendations, assessing their feasibility and benefits. This study of emotional perinatal pathways of 100 couples highlighted the importance and feasibility of screening all patients, with or without risk factors, at different key time points demonstrating undeniable benefits in depression screening, uncovering cases that would have otherwise gone unnoticed. Risk factors for each time point are sometimes commune but also specific of each perinatal period.

In future research, it will be imperative to extend these screening procedures to verify their effectiveness in a larger population. This will require increasing the size of the cohort and replicating the screening process, as we have done, consistently at three key points: during the 9th month of pregnancy, at birth and during the postnatal period. Such an approach would increase the likelihood of identifying all individuals at risk.

In addition, there is an urgent need to raise awareness of partners’ depression among professionals and adapt the screening tool for them. This will enable to better support the both partners in this difficult phase and also highlight the often overlooked risks associated with paternal perinatal depression.

## References

[1] Shorey S, Chee CYI, Ng ED, Chan YH, Tam WWS, Chong YS. Prevalence and incidence of postpartum depression among healthy mothers: a systematic review and meta-analysis. J Psychiatr Res. 2018;104:235–48. doi:10.1016/j.jpsychires.2018.08.001. 30114665

[2] Les morts maternelles en France : mieux comprendre pour mieux prévenir. 6e rapport de l’Enquête Nationale Confidentielle sur les Morts Maternelles (ENCMM), 2013‐2015. Saint‐Maurice : Santé publique France, janvier 2021. 7 p. Available from URL: www.santepubliquefrance.fr et http://www.xn--epop-inserm-ebb.fr/grandes-enquetes/enquete-nationale-confidentielle-sur-les-morts-maternelles.

[3] Slomian J, Honvo G, Emonts P, Reginster J-Y, Bruyère O. Consequences of maternal postpartum depression: a systematic review of maternal and infant outcomes. Women’s Health (Lond). 2019;15:174550651984404. doi:10.1177/1745506519844044. PMC6492376 31035856

[4] Kasamatsu H, Tsuchida A, Matsumura K, Shimao M, Hamazaki K, Inadera H, et al. Understanding the relationship between postpartum depression one month and six months after delivery and mother-infant bonding failure one-year after birth: results from the Japan environment and Children’s study (JECS). Psychol Med. 2020;50(1):161–9. doi:10.1017/S0033291719002101. 31474232 PMC6945325

[5] Giallo R, Woolhouse H, Gartland D, Hiscock H, Brown S. The emotional–behavioural functioning of children exposed to maternal depressive symptoms across pregnancy and early childhood: a prospective Australian pregnancy cohort study. Eur Child Adolesc Psychiatry. 2015;24(10):1233–44. doi:10.1007/s00787-014-0672-2. 25572869

[6] Meaney MJ. Perinatal maternal depressive symptoms as an issue for population health. AJP. 2018;175(11):1084–93. doi:10.1176/appi.ajp.2018.17091031. 30068258

[7] Guintivano J, Sullivan PF, Stuebe AM, Penders T, Thorp J, Rubinow DR, et al. Adverse life events, psychiatric history, and biological predictors of postpartum depression in an ethnically diverse sample of postpartum women. Psychol Med. 2018;48(7):1190–200. doi:10.1017/S0033291717002641. 28950923 PMC6792292

[8] Robertson E, Grace S, Wallington T, Stewart DE. Antenatal risk factors for postpartum depression: a synthesis of recent literature. Gen Hosp Psychiatry. 2004;26(4):289–95. doi:10.1016/j.genhosppsych.2004.02.006. 15234824

[9] Guintivano J, Manuck T, Meltzer-Brody S. Predictors of postpartum depression: a comprehensive review of the last decade of evidence. Clin Obstet Gynecol. 2018;61(3):591–603. doi:10.1097/GRF.0000000000000368. 29596076 PMC6059965

[10] Savelon SV. Accoucher en contexte de pandémie Covid-19, entre isolement et confinement, un temps suspendu pour les dyades et les triades? Le Carnet PSY 2021/1 (N° 240). 2021;1:22–5.

[11] Scarff JR. Postpartum depression in men. Innov Clin Neurosci. 2019;16(5):4. PMC6659987 31440396

[12] Paulson JF, Bazemore SD. Prenatal and postpartum depression in fathers and its association with maternal depression: a meta-analysis. JAMA. 2010;303(19):1961. doi:10.1001/jama.2010.605. 20483973

[13] Smythe KL, Petersen I, Schartau P. Prevalence of perinatal depression and anxiety in both parents: a systematic review and meta-analysis. JAMA Netw Open. 2022;5(6):e2218969. doi:10.1001/jamanetworkopen.2022.18969. 35749112 PMC9233234

[14] Cox JL, Holden JM, Sagovsky R. Detection of postnatal depression: development of the 10-item Edinburgh Postnatal Depression Scale. Br J Psychiatry. 1987;150(6):782–6. doi:10.1192/bjp.150.6.782. 3651732

[15] Rubertsson C, Börjesson K, Berglund A, Josefsson A, Sydsjö G. The Swedish validation of Edinburgh Postnatal Depression Scale (EPDS) during pregnancy. Nord J Psychiatry. 2011;65(6):414–18. doi:10.3109/08039488.2011.590606. 21728782

[16] *Loi n° 2019–1446 du 24 décembre 2019 de financement de la sécurité sociale pour 2020, article 62; Paris; France*

[17] *Loi n° 2021–1754 du 23 décembre 2021 de financement de la sécurité sociale pour 2022, article 86; Paris; France*

[18] Johnson TEL, Clare CA, Johnson JE, Simon MA. Preventing perinatal depression now: A call to action. J Women’s Health. 2020;29(9):1143–7. doi:10.1089/jwh.2020.8646. PMC7520910 32749917

[19] Matthey S, Barnett B, Kavanagh DJ, Howie P. Validation of the Edinburgh Postnatal Depression Scale for men, and comparison of item endorsement with their partners. J Affect Disord. 2001;64(2–3):175–84. doi:10.1016/S0165-0327(00)00236-6. 11313084

[20] Figueiredo B, Conde A. Anxiety and depression in women and men from early pregnancy to 3-months postpartum. Arch Womens Ment Health. 2011;14(3):247–55. doi:10.1007/s00737-011-0217-3. 21479759

[21] Taylor A, Atkins R, Kumar R, Adams D, Glover V. A new mother-to-infant bonding scale: links with early maternal mood. Arch Womens Ment Health. 2005;8(1):45–51. doi:10.1007/s00737-005-0074-z. 15868385

[22] Gawlik S, Julaidan GS, Alsuwaylimi RA, Almajed BM, AlShammari RT, AlFirm RB, et al. Prevalence of paternal perinatal depressiveness and its link to partnership satisfaction and birth concerns. Arch Womens Ment Health. 2014;17(1):49–56. doi:10.1007/s00737-013-0377-4. 24022743

[23] Préparation à la naissance et à la parentalité, recommandations professionnelles, Haute Autorité de Santé (HAS), 2005, Paris. Available from URL: https://www.has-sante.fr/upload/docs/application/pdf/preparation_naissance_recos.pdf.

[24] Guedeney N, Fermanian J. Validation study of the French version of the Edinburgh Postnatal Depression Scale (EPDS): new results about use and psychometric properties. Eur Psychiatry. 1998;13(2):83–9. doi:10.1016/S0924-9338(98)80023-0. 19698604

[25] Lakkis NA, Mahmassani DM. Screening instruments for depression in primary care: a concise review for clinicians. Postgrad Med. 2015;127(1);99–106. doi:10.1080/00325481.2015.992721. 25526224

[26] Peng S, Lai X, Du Y, Meng L, Gan Y, Zhang X. Prevalence and risk factors of postpartum depression in China: a hospital-based cross-sectional study. J Affect Disord. 2021;282:1096–100. doi:10.1016/j.jad.2021.01.012. 33601683

[27] AUDIPOG, Université Cl. Bernard Lyon 1 - Laënnec7, rue Guillaume Paradin - 69372 Lyon Cedex 08, Available from URL: https://www.audipog.net/Courbes-morpho

[28] Pataky EA and Ehlert U. Longitudinal assessment of symptoms of postpartum mood disorder in women with and without a history of depression. Arch Womens Ment Health. 2020;23(3):391–9. doi:10.1007/s00737-019-00990-4. 31350668

[29] Orsolini L, Valchera A, Vecchiotti R, Tomasetti C, Iasevoli F, Fornaro M, et al. Suicide during perinatal period: epidemiology, risk factors, and clinical correlates. Front Psych. 2016;7:138. doi:10.3389/fpsyt.2016.00138. PMC4981602 27570512

[30] Cameron EE, Sedov ID, Tomfohr-Madsen LM. Prevalence of paternal depression in pregnancy and the postpartum: an updated meta-analysis. J Affect Disord. 2016;206:189–203. doi:10.1016/j.jad.2016.07.044. 27475890

[31] O’Brien AP, McNeil KA, Fletcher R, Conrad A, Wilson AJ, Jones D, et al. New fathers’ perinatal depression and anxiety—treatment options: an integrative review. Am J Mens Health. 2017;11(4):863–76. doi:10.1177/1557988316669047. 27694550 PMC5675308

[32] Duchesne S, Donnadieu A-C, Chariot P, Louis-Sylvestre C. Screening for domestic violence during pregnancy follow-up: evaluation of an intervention in an antenatal service. Arch Womens Ment Health. 2021;24(2):293–301. doi:10.1007/s00737-020-01058-4. 32951079

[33] Ramiro-Cortijo D, Soto-Balbuena C, Rodríguez-Muñoz MF. Early association factors for depression symptoms in pregnancy: a comparison between Spanish women spontaneously gestation and with assisted reproduction techniques. JCM. 2021;10(23):5672. doi:10.3390/jcm10235672. 34884374 PMC8658584

[34] Lagadec N, Steinecker M, Kapassi A, Magnier AM, Chastang J, Robert S, et al. Factors influencing the quality of life of pregnant women: a systematic review. BMC Pregnancy Childbirth. 2018;18(1):455. doi:10.1186/s12884-018-2087-4. 30470200 PMC6251086

[35] Monti F, Agostini F, Fagandini P, La Sala GB, Blickstein I. Depressive symptoms during late pregnancy and early parenthood following assisted reproductive technology. Fertil Steril. 2009;91(3):851–7. doi:10.1016/j.fertnstert.2008.01.021. 18314111

[36] Hammarberg K, Fisher JRW, Wynter KH. Psychological and social aspects of pregnancy, childbirth and early parenting after assisted conception: a systematic review. Hum Reprod Update. 2008;14(5):395–414. doi:10.1093/humupd/dmn030. 18653674

[37] Capuzzi E, Caldiroli A, Ciscato V, Zanvit FG, Bollati V, Barkin JL, et al. Is in vitro fertilization (IVF) associated with perinatal affective disorders? J Affect Disord. 2020;277:271–8. 10.1016/j.jad.2020.08.006. 32841828

[38] Gressier F, Letranchant A, Cazas O, Sutter-Dallay AL, Falissard B, Hardy P. Post-partum depressive symptoms and medically assisted conception: a systematic review and meta-analysis. Hum Reprod. 2015;30(11):2575–86. doi:10.1093/humrep/dev207. 26345689

[39] Gambadauro P, Iliadis S, Bränn E, Skalkidou A. Conception by means of in vitro fertilization is not associated with maternal depressive symptoms during pregnancy or postpartum. Fertil Steril. 2017; 108(2):325–32. doi:10.1016/j.fertnstert.2017.06.006. 28651958

[40] de Souza VCA, Parlato-Oliveira E, Anchieta LM, Machado AMC, Savelon SV. The effects of prenatal diagnosis on the interaction of the mother–infant dyad: a longitudinal study of prenatal care in the first year of life. Front Psychol. 2022;13:804724. doi:10.3389/fpsyg.2022.804724. 35418908 PMC8996076

[41] Martínez-Galiano JM, Hernández-Martínez A, Rodríguez-Almagro J, Delgado-Rodríguez M, Gómez-Salgado J. Relationship between parity and the problems that appear in the postpartum period. Sci Rep. 2019;9(1):11763. doi:10.1038/s41598-019-47881-3. 31409871 PMC6692385

[42] Dubey A, Chatterjee K, Chauhan VS, Sharma R, Dangi A, Adhvaryu A. Risk factors of postpartum depression. Ind Psychiatry J. 2021 Oct;30(Suppl 1):S127–S131. doi:10.4103/0972-6748.328803. Epub 2021 Oct 22. PMID: 34908678; PMCID: PMC8611548. 34908678 PMC8611548

[43] Iwata H, Mori E, Sakajo A, Aoki K, Maehara K, Tamakoshi K. Prevalence of postpartum depressive symptoms during the first 6 months postpartum: association with maternal age and parity. J Affect Disord. 2016;203:227–32. doi:10.1016/j.jad.2016.06.002. 27295378

[44] Aurélie B, Catherine G, Claire M, et al. « L’entretien prénatal précoce : des mots sur des maux », Spirale, 2013/2 (N° 66), p. 163–175. doi:10.3917/spi.066.0163. URL:https://www.cairn.info/revue-spirale-2013-2-page-163.htm

[45] Odent M. New reasons and new ways to study birth physiology. Int J Gynecol Obstet. 2001;75:S39–45. doi:10.1016/S0020-7292(01)00512-4. 11742641

[46] Moura D, Canavarro MC, Figueiredo-Braga M. Oxytocin and depression in the perinatal period—a systematic review. Arch Womens Ment Health. 2016;19(4):561–70. doi:10.1007/s00737-016-0643-3. 27295067

[47] Zhao X, Zhang Z. Risk factors for postpartum depression: an evidence-based systematic review of systematic reviews and meta-analyses. Asian J Psychiatr. 2020;53:102353. doi:10.1016/j.ajp.2020.102353. 32927309

[48] Urbanová E, Škodová Z, Bašková M. The association between birth satisfaction and the risk of postpartum depression. IJERPH. 2021;18(19):10458. doi:10.3390/ijerph181910458. 34639758 PMC8508559

[49] Grisbrook M-A, Dewey D, Cuthbert C, McDonald S, Ntanda H, Giesbrecht GF, et al. Associations among caesarean section birth, post-traumatic stress, and postpartum depression symptoms. IJERPH. 2022;19(8):4900. doi:10.3390/ijerph19084900. 35457767 PMC9025262

[50] Patel RR, Murphy DJ, Peters TJ for ALSPAC. Operative delivery and postnatal depression: a cohort study. BMJ. 2005;330:879. 15734748 10.1136/bmj.38376.603426.D3 PMC556158

[51] Kaya L, Çiğdem Z. Delivery and postpartum depression. J. Educ. Health Promot. 2019;8:6. 30815476 10.4103/jehp.jehp_97_18 PMC6378817

[52] Clesse C, Cottenet J, Lighezzolo-Alnot J, Goueslard K, Scheffler M, Sagot P, et al. Episiotomy practices in France: epidemiology and risk factors in non-operative vaginal deliveries. Sci Rep. 2020;10(1):20208 doi:10.1038/s41598-020-70881-7. 33214621 PMC7677317

[53] Ducarme G, Pizzoferrato AC, de Tayrac R, Schantz C, Thubert T, Le Ray C, et al. Perineal prevention and protection in obstetrics: CNGOF clinical practice guidelines. J Gynecol Obstet Hum Reprod. 2019;48(7):455–60. doi:10.1016/j.jogoh.2018.12.002. 30553051

[54] Carroli G, Mignini L. Episiotomy for vaginal birth. In: The Cochrane Collaboration, editor. Cochrane database of systematic reviews. Chichester: John Wiley & Sons, Ltd; 2009, p. CD000081. doi:10.1002/14651858.CD000081.pub2. PMC4175536 19160176

[55] Leeman LM, Rogers RG. Sex after childbirth: postpartum sexual function. Obstet Gynecol. 2012;119(3):647–55. doi:10.1097/AOG.0b013e3182479611. 22353966

[56] Séjourné N, De la Hammaide M, Moncassin A, O’Reilly A, Chabrol H. Étude des relations entre la douleur de l’accouchement et du post-partum, et les symptômes dépressifs et traumatiques. Gynecol Obstet Fertil Senol. 2018;46(9):658–63. doi:10.1016/j.gofs.2018.06.002. 29933918

[57] Boudou M, Séjourné N, Chabrol H. Douleur de l’accouchement, dissociation et détresse périnatales comme variables prédictives de symptômes de stress post-traumatique en post-partum. Gynecol Obstet Fertil. 2007;35(11):1136–42. doi:10.1016/j.gyobfe.2007.09.014. 17996476

[58] Whitburn LY. Labour pain: from the physical brain to the conscious mind. J Psychosom Obstet Gynecol. 2013;34(3):139–43. doi:10.3109/0167482X.2013.829033. 23952172

[59] Collège national des universitaires en psychiatrie, Association pour l’enseignement de la sémiologie psychiatrique, and Collège universitaire national des enseignants en addictologie, editors. Référentiel de psychiatrie et addictologie: Psychiatrie de l’adulte, psychiatrie de l’enfant et de l’adolescent, addictologie. 2e éd. in L’officiel ECN. Tours: Presses universitaires François-Rabelais; 2016.

[60] Denis A, Callahan S. État de stress post-traumatique et accouchement classique: Revue de littérature. Journal de Thérapie Comportementale et Cognitive, 2009;19(4):116–19. doi:10.1016/j.jtcc.2009.10.002.

[61] Liu C, Butwick A, Sand A, Wikström A-K, Snowden JM, Stephansson O. The association between postpartum hemorrhage and postpartum depression: a Swedish national register-based study. PLoS One. 2021;16(8):e0255938. doi:10.1371/journal.pone.0255938. 34379698 PMC8357098

[62] Miller LJ. Postpartum depression. JAMA. 2002;287(6):762–5. doi:10.1001/jama.287.6.762. 11851544

